# Rituximab for Immune Checkpoint Inhibitor Myasthenia Gravis

**DOI:** 10.7759/cureus.16337

**Published:** 2021-07-12

**Authors:** Neha Verma, Muhammad Jaffer, Yolanda Pina, Edwin Peguero, Sepideh Mokhtari

**Affiliations:** 1 Internal Medicine, Moffitt Cancer Center, Tampa, USA; 2 Neurology, Universty of South Florida Morsani College of Medicine, Tampa, USA; 3 Neuro-Oncology, Moffitt Cancer Center, Tampa, USA; 4 Neurology, Moffitt Cancer Center, Tampa, USA

**Keywords:** nivolumab, ipilmumab, melanoma, myasthenia gravis, rituximab

## Abstract

The use of immune checkpoint inhibitors (iCPI) in the treatment of multiple cancers has gained prominence due to their high efficacy. However, neurological immune-related adverse events (irAEs) such as myasthenia gravis (MG) have been associated with iCPI therapy. Most of these neurological irAEs are rare, and in many cases, their diagnoses and management can be challenging.

We present a case of a 70-year-old woman with stage IIIC melanoma who developed a new onset of gradually progressive dyspnea, diplopia, and bilateral ptosis following treatment with one cycle of nivolumab and ipilimumab (Nivo+Ipi). She was diagnosed with MG via positive serum acetylcholine receptor (AChR) antibodies. She had developed a severe dyspnea at rest, which was refractory to multiple immune-suppressive therapies including prednisone, pyridostigmine, and intravenous immunoglobulin (IVIG). Subsequently, she was treated with rituximab 375 mg/m^2^ monthly every four weeks with significant improvement of her symptoms within 48 hours each time.

As the implementation of immunotherapy increases in medical practice, irAEs may become more apparent. When first-line therapies are not adequate, other alternative therapies should be explored. This case of MG as an irAE shows that rituximab can provide a potential benefit to treating patients with immunotherapy-induced MG who are refractory to other standard treatments. Prospective studies are needed to further evaluate the efficacy of rituximab in the management of irAEs.

## Introduction

Immune checkpoint inhibitors (iCPI) have gained prominence in the treatment of a wide variety of cancers. Nivolumab (Nivo) is a monoclonal antibody to the programmed cell death protein-1 (PD-1) receptor and ipilimumab (Ipi) is an inhibitor of the cytotoxic T-lymphocyte-associated protein 4 (CTLA-4)-associated antigen. Both Nivo and Ipi (Nivo + Ipi) inhibit cellular signals responsible for the downregulation of the T-cell response, allowing upregulation of the immune response to target cancer cells [1]. However, several neurological immune-related adverse events (irAEs) have been associated with iCPI use, including myasthenia gravis (MG) among other rare neurological syndromes [2-10]. The incidence of MG is 0-0.24% with iCPI [11]. These syndromes are rare and difficult to diagnose, and a full neurological evaluation should not be delayed. In addition, given their rarity, their pathophysiology remains unknown and treatment options are limited to current irAE guidelines [1].

Rituximab, a monoclonal antibody to CD20, is not currently part of the guidelines to manage treatment-resistant iCPI-induced MG. The only suggested treatments include pyridostigmine, corticosteroids, intravenous immunoglobulin (IVIG), and plasmapheresis [1,12]. iCPI-induced MG is a rare, emerging toxicity with recent literature estimating a mortality rate of approximately 30% [13]. We present here the second case reported in the literature for which rituximab was successfully used for iCPI-induced MG [14,15]. However, this is the first case to document the long-term benefit of Rituximab at 375 mg/kg monthly over 14 months before the patient deceased.

The article was previously published in the ResearchSquare preprint server (https://www.researchsquare.com/article/rs-91621/v1).

## Case presentation

A 70-year-old woman with stage III C melanoma developed new-onset dyspnea, diplopia, and bilateral ptosis two weeks following her first cycle with Nivo + Ipi. She was evaluated by a neurologist and was found to have elevated serum acetylcholine receptor (AChR) binding, modulating, and blocking antibodies. Her symptoms gradually worsened over the course of three cycles of Nivo + Ipi, and the iCPI were discontinued.

**Figure 1:**
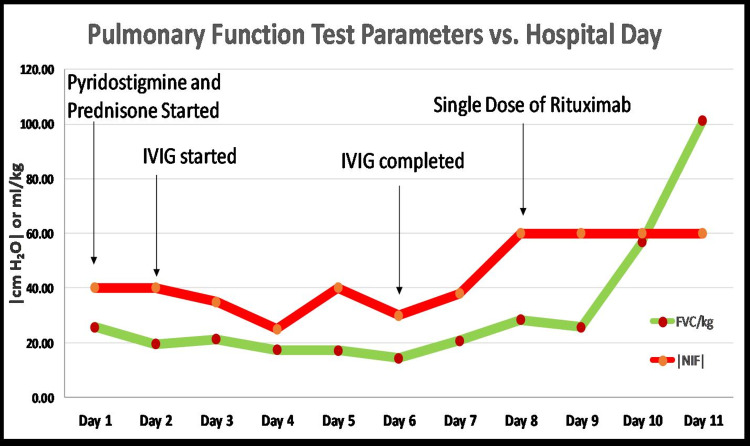
Pulmonary function parameters with forced vital capacity and negative inspiratory force and treatments for irAEs The pulmonary function parameters throughout the hospital visit are shown with forced vital capacity (FVC) and negative inspiratory force (NIF). The patient was initially treated with pyridostigmine 60 mg four times a day and prednisone 90 mg daily, followed by IVIG 2 mg/kg (divided into five days). Her FVCs were consistently low at around 20 mL/kg for eight days including two days after completion of IVIG. Subsequently, she received treatment with a single infusion of rituximab IV 375 mg/m^2^ and within one day post-treatment, her FVCs started to show a marked improvement reaching >100 mL/kg at day 11. There is a significant improvement in the patient’s FVCs and stabilization of her NIF after the administration of a single dose of rituximab IV 375 mg/m^2^. NIF was taken as an absolute value function with normal values <−60 cm H_2_O. Abbreviations: IVIG: intravenous immunoglobulin; FVC/kg: forced vital capacity/ideal body weight in kilograms; NIF: negative inspiratory.

**Figure 2:**
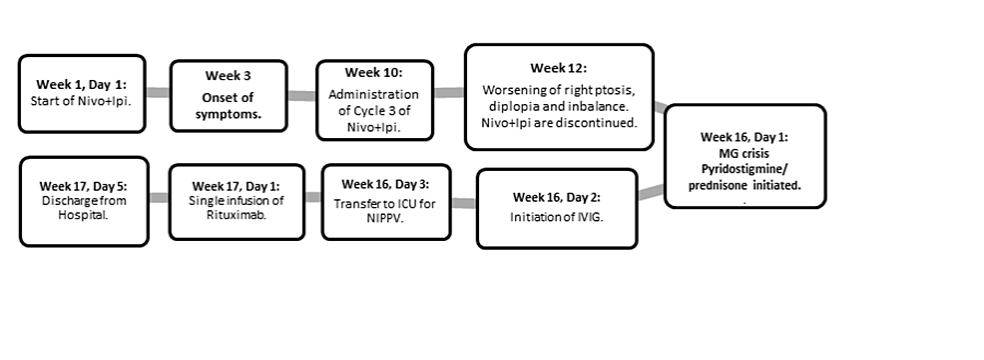
Summary timeline of treatment and clinical symptoms The patient developed MG-like symptoms two weeks following cycle 1 of Nivo + Ipi. Her symptoms gradually progressed and Nivo + Ipi were discontinued after cycle 3. She was found to be in an MG crisis on week 16. She was sequentially treated with pyridostigmine 60 mg four times a day and Prednisone 90 mg daily on week 16 and IVIG 2 g/kg (divided into five days) on week 16 day 2 with no objective improvement in her pulmonary function tests, which were consistently low at 20 mL/kg. She was transferred to the ICU for NIPPV and received treatment with a single infusion of rituximab IV 375 mg/m^2^ on week 17 day 1 with significant improvement in her symptoms including pulmonary function tests showing >100 mL/kg. She was eventually discharged from the hospital on week 17 day 5. Abbreviations: Nivo + Ipi = Nivolumab and Ipilimumab; IVIG: intravenous immunoglobulin; NIPPV: non-invasive positive pressure ventilation.

The patient was referred to the Neuro-Oncology clinic at H. Lee Moffitt Cancer Center (MCC) and Research Institute for further evaluation. The AChR antibodies were further elevated compared to a month prior. Creatine phosphokinase (CPK) level was normal. She was found to have a critically low respiratory function with dyspnea at rest and was admitted to the hospital for further management. She was started on pyridostigmine 60 mg four times a day and Prednisone 90 mg daily. Given her worsening pulmonary function, she was transferred to an intensive care unit and placed on non-invasive positive pressure ventilation (NIPPV). Forced vital capacity was consistently low at approximately 20 mL/kg. On hospital day 2, she was started on a five-day course of IVIG 0.4 gm/kg/day. The patient’s subjective symptoms resolved, but her respiratory function tests continued to decline. On hospital day 8, she received a single dose of rituximab IV 375 mg/m^2,^ and her forced vital capacity dramatically improved within 48 hours, as illustrated in Figure 1. Her respiratory function continued to significantly improve, and she was subsequently discharged from the hospital. A summary of the key events of the case is shown in Figure 2. 

The patient developed worsening of her MG symptoms three to four weeks after the initial rituximab infusion; therefore, she was treated with a total of 14 more cycles of a single dose of rituximab 375 mg/m^2^ on a monthly basis, before she succumbed to her metastatic melanoma.

## Discussion

Ever since their initial approval by the Federal Drug Administration (FDA) for the treatment of unresectable metastatic melanoma in 2015, the use of iCPI has rapidly expanded to include many other cancers [15]. There are a number of irAEs that have been associated with iCPI use, including those that produce rare and difficult to diagnose and treat neurological conditions [2-10]. For example, multiple cases of MG associated with iCPI have been reported in the literature [2,5,6]. 

Guidelines for the treatment of iCPI associated MG, include the use of pyridostigmine, corticosteroids, IVIG, and plasmapheresis [1,12,16]. However, some patients are refractory to these treatments. Rituximab has been previously reported as a potential third-line therapy for non-iCPI-related MG which is refractory to standard immunosuppressive approaches [15]. To the best of our knowledge, our patient is the second case reported with iCPI-induced MG with a treatment benefit with rituximab [14].

Our patient showed a dramatic improvement in her pulmonary function tests and other MG-related symptoms (dyspnea, diplopia, and ptosis) immediately after the initial therapy with rituximab IV 375 mg/m^2^, as well as following every rituximab infusion thereafter. In this case, rituximab therapy had lasting therapeutic effects overall for three to four weeks. The optimal schedule of rituximab is unclear and published experience suggests a 375 mg/m^2^ weekly × four doses in the NCCN guidelines [17]. Nonetheless, our patient showed a marked clinical and objective improvement on a single dose given monthly. This could be explained by the half-life of the drug. Her clinical and objective response to rituximab was consistent with the response typically seen in patients with non-ICI-related MG [16].

It is also possible that her clinical improvement was due to her other MG treatments, which are pyridostigmine, prednisone, and IVIG therapy, which were administered two days prior to rituximab. Certainly, her favorable clinical response to rituximab could have been secondary to a delayed effect of these standard MG therapies. However, the close temporal association between clinical improvement and each rituximab infusion as well as the dramatic objective response within 48 hours of rituximab administration suggests that rituximab treatment produced her dramatic recovery.

Although rituximab traditionally acts to deplete the plasma cells, recent evidence has shown that it has a direct influence on the T-cell responses [18]. Given this common substrate with iCPI, perhaps rituximab should be considered as a more effective therapy in iCPI-induced MG compared to the traditional and standard immunosuppressive therapies [1,12,16]. We have not found any literature or clinical trials to look at rituximab for the treatment of iCPI-induced MG but our institution is considering conducting this trial. It is important to report similar successful cases in the literature to increase awareness about these rare neurological irAEs and make others acquainted with the use of non-standard therapies in cases that are refractory to standard therapy. It is also important to obtain a prompt neurological consultation as soon as these types of irAEs are suspected, as these syndromes are difficult to detect and could be misdiagnosed. We suggest considering a low dose of rituximab (375 mg/m^2^) as an initial therapeutic option for iCPI-induced MG that is refractory to standard immunotherapies. This might help to achieve a quicker positive clinical response, avoid unnecessary hospitalizations, and the serious side effects of chronic corticosteroid use.

As with any case report, there are limitations to consider. We have interpreted this patient’s MG to be de novo and directly related to iCPI administration. An alternative explanation is that the patient had preexisting asymptomatic MG (i.e., with positive AChR antibodies) and treatment with Nivo + Ipi unmasked her condition. In the current literature, the median time from ICPI initiation until the first MG symptom was four weeks (range 6 days to 16 weeks) [11]. Also, while we observed an excellent and reproducible response to rituximab, prospective studies will be needed to definitively evaluate the efficacy of rituximab in iCPI-induced MG. Establishing an animal model of iCPI-associated MG may also suggest mechanistic and therapeutic interventions. However, given the rarity of this condition, including in both non-immunotherapy and immunotherapy-induced cohorts, it may be challenging to complete prospective clinical studies needed to prove the efficacy of this approach. Further evaluation with prospective studies looking at AChR antibodies pre and post-iCPI treatment may be useful to help establish the biological basis for iCPI-induced MG.

## Conclusions

This case report suggests that rituximab may be an effective alternative or addition to the standard treatments for iCPI-induced MG refractory to current standard immunosuppressive therapies. As the use of iCPI is rapidly increasing to treat a variety of malignancies, more neurological irAEs are expected to occur, including rare neurological conditions that are difficult to manage and can be resistant to current treatments per AE guidelines. Future prospective clinical studies regarding the safety profile, optimal dosing, and effectiveness of rituximab in patients with iCPI-induced MG present an exciting route of inquiry.
